# An assessment of responses to egg production and liver health of Japanese quails subjected to different levels of metabolizable energy

**DOI:** 10.5713/ab.22.0095

**Published:** 2022-09-02

**Authors:** Diana Maryuri Correa Castiblanco, Michele Bernardino de Lima, Silvana Martinez Baraldi Artoni, Erikson Kadoshe de Morais Raimundo, Daniel Silva Santos, Lizia Cordeiro de Carvalho, Edney Pereira da Silva

**Affiliations:** 1 Department of Animal Sciences, Universidade Estadual Paulista, Jaboticabal, São Paulo, 14884900, Brazil

**Keywords:** Egg Mass, Egg Weight, Hepatic Physiology, Oviduct Morphometry, Quail Production

## Abstract

**Objective:**

Current quail production is configured as an economic activity in scale. Advancements in quail nutrition have been limited to areas such as breeding and, automation of facilities and ambience. The objective of this study was to evaluate the performance responses, liver and oviduct morphometry, and liver histology of Japanese laying quails subjected to different levels of nitrogen-corrected apparent metabolizable energy (MEn).

**Methods:**

A completely random design was used that consisted of nine levels of MEn, six replicates, and five hens per cage with a total of 270 quails. The experimental period lasted for 10 weeks. The variables of performance were subjected to analysis of variance and then regression analysis using the broken-line model. The morphometric and histological variables were subjected to multivariate exploratory techniques.

**Results:**

The MEn levels influenced the responses to zootechnical performance. The broken-line model estimated the maximum responses for feed intake, egg production, egg weight, and egg mass as 3,040, 2,820, 1,802, and 2,960 kcal of MEn per kg of diet, respectively. Multivariate analysis revealed that the occurrence of hepatic steatosis and increased levels of Kupffer cells were not related to MEn levels.

**Conclusion:**

The level of 2,960 kcal/kg of MEn meets performance variable requirements without compromising hepatic physiology.

## INTRODUCTION

Advances in genetics have allowed for the segregation of the multiplication and production sectors, and this is indicative of the degree of professionalization that the quail egg industry has achieved in recent decades. The facilities were adapted for Japanese quails, and currently, sheds possessing temperature control automation, dung removal, and egg collection are present at the main commercial egg-producing locations [1–3].

Attention should be focused on the concentration of nitrogen-corrected apparent metabolizable energy (MEn) in the diet. Therefore, the concentration of MEn in the diet should be established based on certain criteria. However, publications during the last two decades do not show a consensus regarding the amount of MEn required. Here, it is understood that a reduction in the difference between the values recommended in the literature is necessary. We located 11 studies examining MEn for quail hens, and the recommendations ranged from 2,600 to 3,100 kcal/kg and egg production (EP) ranged from 77% to 94% using Japanese and European quails [4–14]. When analyzing these publications, a common theme was that the mean concentration of MEn in the diet was 2,876 kcal/kg with an amplitude ranging from 87% to 110%. The lowest MEn tested was 2,500 kcal/kg [11]. The association of narrow amplitude with the *ad libitum* feed intake (FI) option may have been an attenuating factor in the imposed deficiency and, consequently, in the absence of an effect of the MEn levels tested in most of the studies reviewed here.

The possibility of obtaining a greater amplitude of the MEn values in the experimental diets may involve the use of the dilution technique; however, this is a common practice in amino acid studies [15]. For MEn, only one study using commercial laying hens utilized this technique [15], and with quails, the use of this technique is nonexistent. Another missing description in the literature is the impact of the concentration of MEn on hepatic physiology and oviduct morphometry of current Japanese quail lines improved for commercial posture. For other nutrients such as protein, the possible effects have already been described [16]. Thus, in this study we investigated the performance responses, liver and oviduct morphometry, and hepatic histology of laying Japanese quails subjected to different levels of MEn.

## MATERIALS AND METHODS

The Animal Ethics and Welfare Committee of Universidade Estadual Paulista approved all experimental procedures used in this study (protocol number 6.725/15).

### Birds and husbandry housing

Two hundred and seventy female Japanese quail (VICAMI strain at 16 weeks of age) were housed in galvanized wire cages measuring 1.0 m×0.5 m×0.15 m. The cages were equipped with feeders and nipple drinkers in a climatic chamber at room temperature. The birds were selected and uniformly distributed in the experimental units based on their weight (185±7 g) and EP (78%±6%). The light program that was used was a 16 L:8 D. The minimum, average, and maximum temperatures were 18°C, 22°C, and 26°C, respectively. The minimum, average, and maximum humidity values were 50%, 60%, and 68%, respectively. The experiment lasted ten weeks. The first four weeks involved adaptation, and the last six weeks were for data collection. All birds were fed 27 g of the diet per day and were supplied twice each day (morning and afternoon).

### Experimental diets

The treatments were distributed in a completely randomized design that consisted of nine increasing levels of MEn (1,609, 1,740, 1,895, 2,260, 2,394, 2,643, 2,892, 3,045, and 3,058 kcal/kg, respectively) with six replicates of five quails per experimental unit. Two diets were formulated with high and low MEn contents (Table 1) according to previously published recommendations [17] (Table 2).

The high MEn diet was formulated to contain 114% greater than the MEn recommendation (2,850 kcal/kg), and the low MEn diet was formulated to contain approximately 52% less than the MEn recommendation. The remaining samples were obtained by dilution (Table 3). Dietary protein, other nutrients, amino acids, vitamins, and minerals were maintained at constant levels so as not to be limiting.

### Measurements of responses

Egg production (EP, %) was recorded daily, and egg weight (EW, g) was measured on three consecutive days each week. Egg mass (EM, g/bird·d) was determined using EP and EW. Feed leftovers were weighed weekly to quantify the weekly FI (g/bird·d). The feed conversion ratio (FCR, g/g) was calculated by dividing FI by EM and was corrected for mortality. Body weight (BW, g/bird) was measured at the first and tenth week of the assay. The crude fiber (CF) content of the feed or its indigestible portion was used as a measure of physical capacity intake or scaled feed intake (SFI, g/kg metabolic BW^0.67^).

At the end of the tenth week, excreta were collected using the total collection method to determine the MEn of the experimental diets. Ferric oxide (10 g/kg) was added to each treatment diet with proper mixing as a marker to define the beginning and end of the excreta collection period. The period of adaptation to diet was 72 h. The excreta were collected in adapted trays under the cages twice each day for three days, packed in plastic bags daily, and stored in a freezer (−20°C) until the end of the collection period when the samples were then homogenized per experimental unit, pre-dried, and ground in a ball mill. The diets and excreta samples were sent to the Laboratory of Poultry Sciences for analysis of dry matter (AOAC Official Method 930.15), total nitrogen (AOAC Official Method 2001.11), and crude energy (Model Parr 6200 oxygen bomb calorimeter), and MEn values were calculated. The CF of the diet was analyzed on a dosi-fiber machine using the AOAC Official Method 920.39.

### Tissue sampling and euthanasia

At the end of the experiment, 54 birds were selected (one bird per replicate) to analyze the oviduct morphometry and liver histology. The birds were individually identified and euthanized after an eight-hour fasting period. Carbon dioxide was used in a transparent chamber to allow individual birds to be observed. The flow rate was maintained at approximately 20% of the chamber volume per min. The gas flow was maintained for at least 3 min after apparent clinical death. The chamber was sanitized after each euthanasia procedure.

The livers and oviducts were collected, measured using an electronic digital device, and weighed on a digital scale (0.001 g). The right and left lobes of the livers were separately weighed. The number of folds in each magnum and isthmus was quantified.

### Physiological responses

For analysis of the histological parameters of the liver, samples from the median region of the left lobe (approximately 1 cm^2^) were collected and fixed immediately in Bouin's solution for 24 h. They were then washed in alcohol (70%) to remove the fixative, dehydrated in a series of alcohols, diaphanized in xylol, and embedded in paraffin. Four semi-serial histological sections (7 μm thickness) were obtained from each bird and placed on the histological slides, and they were subsequently stained using the hematoxylin-eosin (HE) technique. Five fields were photographed randomly from each cut with the aid of a digital camera (Leica DFC 295) attached to a microscope (Leica-DM 2500), and the images were analyzed using LAS V.3.8 (Leica, Wetzlar, Germany).

Three photomicrographs per slide based on a randomization table were randomly chosen to determine variables. The areas of Kupffer cells and steatosis were measured [18]. To count Kupffer cells and steatosis, a grid with five frames (50×50 μm each) composed of dotted and continuous lines was superimposed onto each of the photos to randomize the counting area. Cell counting was performed on all frames (including cells on the dotted lines) while discarding those located on the continuous lines [18].

### Statistical analysis

#### Univariate statistics

The data from the current experiment were analyzed using the PROC NLIN statement of SAS 9.4 (Statistical Analysis for Windows, SAS Institute Inc., Cary, NC, USA). The linear plateau models (one slope and two slopes) were adjusted according to previously described procedures [19].

The responses (ξ) were considered as dependent variables, and the mean levels were analyzed (X) as independent variables. A broken-line model was used with a slope (Eq. 1), quadratic (Eqs. 2), and two slopes or two lines (Eq. 3).


(Eq. 1) ξ=ψ+α×(τ-X)

(Eq. 2) ξ=ψ+α×(τ-X)×(τ-X)

(Eq. 3) ξ=ψ+α×(τ-X)+β×(X-τ)

where (τ–X) is defined as zero when X>τ for the single-slope Eq. 1 and quadratic Eq. 2. The two-slope broken-line model expressed in Eq. 3, (τ–X) is defined as zero at X>τ, and (X–τ) is defined as zero when X<τ. We used the parameters for the breakpoint X value (τ), an asymptote for the first segment (ψ), and slopes for the 2 line segments (α, β). Parameters were estimated using the procedure described previously [19].

#### Multivariate statistics

The morphometric and histological variables were subjected to factorial analysis that is a multivariate exploratory technique. Thirteen variables were used for this analysis, including BW, liver weight, liver length, right liver lobe weight, left liver lobe weight, oviduct weight, oviduct length, number of isthmus folds, number of ovary folds of magnum, number of Kupffer cells, number of steatoses, area of Kupffer cells, and area of steatosis. First, the adequacy of the sample space was analyzed through the correlation matrix, Kaiser-Meyer-Olkin test, Bartlett sphericity test, anti-image matrix, and commonalities [20]. The results led to the exclusion of the weight of the bird, number of folds of the isthmus, and number of Kupffer cells from the factorial analysis. Then, we used principal components as a method to extract the factors while considering that the value 1.00 is the minimum for an eigenvalue to be significant and for the load of a variable to be considered significant. The minimum value was 0.70 within one factor.

Factor analysis was used to create latent variables associated with the originally measured variables to thus allow analysis of variance considering several variables simultaneously to verify the statistical significance of the relationship between the latent variables classified within each factor and its relationship with the levels of MEn. Considering the means of extracting latent variables, we here may refer to multivariate analysis of variance.

## RESULTS

In general, the various MEn levels modified the bird responses (p<0.010) for all variables of zootechnical performance (Table 4). Considering FI, none of the MEn levels were the result of birds consuming the full 27 g/d of feed offered. The level of MEn at 1,609 kcal/kg exhibited the highest FI of 25.7 g/d compared to the level of 3,250 kcal/kg for 23.3 g/d, and this represented a difference of 2.4 g. Comparing the results of FI and metabolizable energy intake (MEI) it was observed that at levels of 1,609, 1,740, and 1,895 kcal/kg, the birds consumed more feed and consumed less energy. For diets possessing a greater amount of fiber and as MEn levels increased, the FI decreased and the MEI increased.

### Physical capacity intake

**Figure 1:**
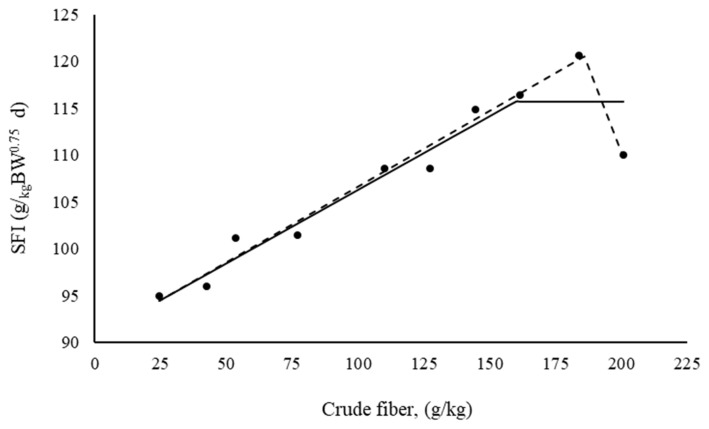
The mean relationship between the scaled feed intake (SFI) of Japanese quails at the laying stage (g feed/kg metabolic body weight/d) and crude fiber content in the feed (g/kg).

According to Figure 1, the broken-line model with two slopes allowed for the description of the SFI that was closer to the mean contour of the treatment responses. The level possessing a higher CF content exhibited a reduction in the SFI. This reduction influenced the response of asymptote shaping when the broken-line model was adjusted with a slope according to the equation:


(4) SFI=116 (±1.86)+0.1567 (±0.029)×[160 (±20.6)-X]

For X<τ and when X>τ, [τ–X] = 0. The reduction in FI as represented by the SFI verified at the level with the highest CF content was explained by the broken-line model with two slopes according to the following equation:


(5) SFI=121 (±0.81)-0.161 (±0.0097)×[186 (±1.99)-X]-0.728 (±0.0043)×[X-186 (±1.99)]

By definition, [186–X] = 0 when X>τ and [X–186] = 0 when X≤τ. The rate of reduction in the SF after 186 g/kg of CF in diet is 4.5 (0.1614/0.7276)-fold greater than is the rate that describes increasing SFI.

### Zootechnical performance

According to the model fitted for FI, the asymptote (ψ) in the FI occurred at 3,040 kcal/kg. According to our model, FI is expected to increase by 0.38% for every 100 kcal reduction in the diet (Table 5).

The lowest EP was 2.3% for the 1,609 kcal/kg level, and the highest EP was 77.8% for the 3,058 kcal/kg level. The asymptote (ψ) for EP was 74%, and this occurred from 2,829 kcal/kg according to the model presented in Table 5. According to this model, a reduction of 100 kcal/kg in the diet resulted in a 7.9% reduction in the EP.

There was a significant difference for the EW (Table 4), where the lowest EW was 10.2 grams (2,260 kcal/kg) and the highest EW was 11.7 g (3,058 kcal/kg). There was a linear increase in EW from 1,802 kcal/kg (Table 4). The two-slope model fitted to this variable is presented in Table 5.

As the birds increased their MEI, there was also an increase in EM. The smallest EM was 0.2 grams (1,609 kcal/kg), and the largest was 9.1 g (3,058 kcal/kg). The response asymptote (ψ) to EM stabilized at 8.44 g/bird·d, and this corresponded to the 2,965 kcal/kg level. According to the model (Table 5), a reduction of 100 kcal/kg in the diet causes the EM to become reduced by 7.0%.

Overall, there was an improvement in FCR as the levels of MEn in the diet increased (Table 4). The highest FCR (98.1± 38.6) was observed for the first level of MEn, and the lowest FCR was observed for the highest level of 3,058 kcal/kg for MEn. The model with a quadratic ascending fit to the variable FCR is presented in Table 5. According to this model, stabilization of the response (ψ) at 3.12 g/g occurred at a concentration of 2,611 kcal/kg.

The BW of the birds at the start of the trial varied from 184 to 188 g (a difference of 3.24 g), and these data are not provided in the tables. At the end of the assay, the weight of the birds varied from 153 to 194 (a 41 g difference) as presented in Table 4. The first four levels of MEn resulted in weight loss in the birds, and the largest losses were observed for the first two levels of MEn (average of 31.76 g). Weight loss decreased the extent to which the MEI was increased. Table 4 indicates a linear increase in the ΔBW of birds that occurred with an increase in the level of MEn in the diet until it reached the point where there was no further response (the response asymptote [ψ]). According to the model (Table 5), the point where the break (τ) occurred was 2,892 kcal/kg, and at this level the ΔBW close to zero (0.007 g).

### Morphometry and histology

The MEn levels did not significantly affect the morphometric variables of the liver and oviduct (Table 6) or the morphological variables of the liver (Table 7). In general, there was a numerical difference between the level of 1,609 kcal/kg and the low average values compared to the 3,059 kcal/kg level. The datasets presented in Tables 6 and 7 were subjected to an analysis of factors to evaluate the interrelationship between the variables (Table 8).

Table 8 presents the four factors obtained in the factorial analysis that was performed for the extracted parameters and the variables comprising each factor. There was no significant effect (p>0.050) for energy level on the parameters that were evaluated by variance analysis (Table 8). The analysis of variables that correlate with each of the factors revealed that factor 1, factor 2, and factor 3 exhibited a positive correlation among a group of variables. Factor 1 indicated that when the weight of the liver was increased, the right lobe weight, left lobe weight, and the number of steatoses increased. Factor 2 indicated that when the weight was increased, the oviduct became increased in length. Factor 3 indicated that when the number of pleats in the magnum was increased, the length of the liver became increased, and factor 4 revealed a negative correlation between Kupffer cells and steatosis cells. This indicated that when the area of the Kupffer cells was increased, the area of the steatosis cells was decreased.

## DISCUSSION

To obtain range of responses using the dose-response method, the evaluated nutrient must be limited. To achieve this, we applied the concept of “theoretical” dilution technique [15]. According to the results obtained for EP and EM, MEn was the most limiting nutritional resource for the validation of the study. This can be verified by the theory of FI regulation that was proposed previously [21] and that states that if the diet is not properly balanced, the bird will increase its FI in an attempt to compensate for the most limiting nutritional resource. We have observed this in our current study as evidenced by the ratio of 0.38% for each reduction of 100 kcal in the diet. This rate is expected to continue to increase. Although it has not been measured, it is speculated that the physicochemical characteristics of the ingredients used in the diluted diet may have attenuated the increase in FI by providing greater filling of the crop.

This physical capacity intake must be measured in terms of feed characteristics such as CF, density, and water-holding capacity, and the appropriate parameter must be measured in feed ingredients [22]. Although the birds used this artifice under adverse nutrient conditions, reaching the level of 121 g/kg of BW^0.67^, was only possible under conditions of term neutrality. This is a condition offered in systems with controlled environments. The recurrence of the use of this ingestion capacity in controlled environments may be related to birds with desirable BW. The quest to regulate FI (the limiting nutrient) inevitably generates excess intake from other routes, and the means by which to store the excess carbon from the constitution of amino acids, fatty acids, and carbohydrates is in the form of body fat. Therefore, it is reasonable to assume that Japanese quail production can regulate FI according to limiting nutrient theory.

An improvement in EP of 2.3% to 77.8% and of EM from 0.2 to 9.1 (g/bird·d) was observed with an increase in the MEn level. This result demonstrates the importance of understanding the nutritional requirements of MEn to achieve the maximum performance potential of these birds. The level of 1,609 kcal/kg presented an EP that was 2.3% lower than that of the treatments, and this explains why the birds were close to the physiological state of maintenance. Moreover, it was observed that 0.57 g of tissue per day was mobilized from body reserve for that purpose. Therefore, the instinct for reproduction was preserved. A similar description was not found in the literature.

Regarding EW, the increasing levels of MEn led to an increase in weight (1.5 g), possibly due to the increase in MEn daily intake being sufficient to meet the production requirements and to obtain heavier eggs. The primary factor that affects EW is BW [1–3]. This was observed in this study, as the birds with slight eggs exhibited a BW loss of 41 g (in the period) compared to that at the levels of 1,609 and 3,050 kcal/kg.

The birds that received diets below 2,394 kcal/kg of MEn presented with FCR due to the MEn dilution of diets and the low EP at those levels. A previous study [5] verified the improvement in FCR with increased MEn. Although in most surveys evaluating the effects of various levels of MEn on quail laying, there have been no observed significant effects in regard to FCR. One factor that can likely explain the absence of effects is the levels that are evaluated, as the lowest level in this study was 1,609 kcal/kg, while in the published works we found that the lowest level was 2,500 kcal/kg [12,13].

The linear increase in the BW of birds can be attributed to the increase in MEI, and this variable has been previously correlated with the variation in BW [1,2]. The results of this research reveal the importance of understanding the energy requirements of Japanese quails during the laying phase.

The levels of MEn used did not influence the morphological parameters of the liver and oviduct or the histology of the liver, thus indicating that levels between 1,609 and 3,050 kcal/kg do not affect the morphometry of the liver or the oviduct. It was expected that birds fed with levels above the recommended levels [23,17] at 2,800 and 2,900 kcal/kg, respectively, would develop steatosis or nutritional diseases such as fatty liver syndrome. A survey reported nutritional disease in Japanese quails but did not report any involvement with MEn levels [24]. However, it has been reported that the relationship between protein and physical activity is a factor that predisposes commercial layers to the appearance of fatty liver [25,26], and quail laying was not determined to support placement based on the literature. Based on this scenario, multivariate analysis techniques were used to maximize relevant data collection. The analysis generated four factors (commonly called processes), and in this study, we refer to these factors as the physiological process. By exploring factor 1 or physiological process contained in factor 1, there is an indication that the increase in the weight of the liver leads to an increase in the weight of the right and left lobe and the area of steatosis, although this increase was not significant. From a physiological point of view, it is expected that this process would be associated with the levels of Men when considering that the liver performs several functions between the storage of carbohydrates and fats [16]. This activity can induce an increase in the weight of the liver (referring to steatosis) and can also be applied based on the knowledge that lipidosis or hepatic steatosis may be derived from several factors ranging from nutritional factors to poisoning cases.

Once the physiological process has been detected, the limitation of this research lies in the attribution of its cause. It is speculated that the lack of significance for a number of our data points was due to the reduced number of birds sampled for this analysis. Therefore, future surveys should use larger sample sizes.

The physiological process contained in factor 2 grouped the variables related to the oviduct, weight, and length, and it exhibited an allometric relation. When the weight was increased, the length became increased. Although they are orthogonal, factor 3 and factor 4 grouped histological variables of the liver demonstrated that when Kupffer cells increase steatosis, this can be explained by the increase in Men. Additionally, the presence of vacuoles in the hepatocytes increases. This association reveals the natural mechanisms used to store energy. Kupffer cells form the major portion of the endothelial reticulum or mononuclear phagocyte system [27], where an excessive increase in steatosis maintains a physiological balance of energy and thus avoids metabolic diseases.

## CONCLUSION

According to the broken-line model, the recommended dietary ME level that maximized egg mass of Japanese laying quails without affecting the liver, oviduct morphometry, and liver histology was 2,960 kcal ME/kg.

## Figures and Tables

**Table 1:** Percentual (%) and nutritional composition as calculated (%) from experimental diets

Items	Low 1,500	Level of metabolizable energy (kcal/kg)							High 3,250

		1,890	2,060	2,230	2,400	2,730	2,960	3,070	
Ingredients									
Corn	16.16	24.95	28.78	32.61	36.44	43.87	49.06	51.53	55.59
Soybean meal 45%	32.91	31.05	30.23	29.42	28.61	27.03	25.94	25.41	24.55
Rice husk	23.11	17.96	15.71	13.47	11.22	6.87	3.83	2.38	-
Soy oil	0.7	2.18	2.83	3.48	4.13	5.38	6.26	6.67	7.36
Lignocellulose 65%	15.00	11.66	10.20	8.74	7.29	4.46	2.49	1.54	-
Limestone	6.72	6.72	6.73	6.73	6.73	6.73	6.74	6.74	6.74
Dicalcium phosphate	1.16	1.16	1.16	1.16	1.16	1.16	1.16	1.16	1.16
Salt	0.28	0.29	0.30	0.31	0.31	0.32	0.33	0.33	0.34
DL-Methionine, 99%	0.86	0.85	0.84	0.84	0.83	0.82	0.82	0.82	0.81
L-Lysine HCl, 78%	0.6	0.63	0.65	0.66	0.68	0.71	0.73	0.73	0.75
L-Arginine	0.72	0.74	0.76	0.77	0.78	0.80	0.81	0.82	0.83
L-Threonine	0.39	0.39	0.40	0.40	0.40	0.40	0.41	0.41	0.41
L-Valine	0.48	0.48	0.48	0.48	0.49	0.49	0.49	0.49	0.49
L-Isoleucine	0.36	0.37	0.37	0.38	0.38	0.39	0.39	0.40	0.40
L-Tryptophan	0.14	0.14	0.15	0.15	0.15	0.15	0.16	0.16	0.16
Vitamin and Mineral premix^1)^	0.40	0.40	0.40	0.40	0.40	0.40	0.40	0.40	0.40
BHT^2)^	0.01	0.01	0.01	0.01	0.01	0.01	0.01	0.01	0.01
Total	100	100	100	100	100	100	100	100	100
Nutritional composition calculated									
Metabolizable energy (kcal/kg)	1,500	1,890	2,060	2,230	2,400	2,730	2,960	3,070	3,250
Crude protein (%)	19.50	19.50	19.50	19.50	19.50	19.50	19.50	19.50	19.50
Digestible lysine (%)	1.30	1.30	1.30	1.30	1.30	1.30	1.30	1.30	1.30
Digestible M+C (%)	1.24	1.24	1.24	1.24	1.24	1.24	1.24	1.24	1.24
Digestible threonine (%)	0.91	0.91	0.91	0.91	0.91	0.91	0.91	0.91	0.91
Digestible tryptophan (%)	0.31	0.31	0.31	0.31	0.31	0.31	0.31	0.31	0.31
Digestible valine (%)	1.13	1.13	1.13	1.13	1.13	1.13	1.13	1.13	1.13
Calcium (%)	2.90	2.90	2.90	2.90	2.90	2.90	2.90	2.90	2.90
Available phosphorus (%)	0.30	0.30	0.30	0.30	0.30	0.30	0.30	0.30	0.30
Sodium (%)	0.14	0.14	0.14	0.14	0.14	0.14	0.14	0.14	0.14

ME, metabolizable energy.

1) Vitamin premix provided the following (per kg of diet): Vit. A 1,750.000 U. I; Vit. D_3_ 500,000 U. I; Vit. E 2,000 U. I; Vit. K_3_ 500 mg; Vit. B_1_ 250 mg; Vit. B_2_ 875 mg; Vit. B_6_ 500 mg; Vit. B_12_ 1,250 mcg/kg; niacin 6,250 mg; choline 65 g; pentatonic acid 2,500 mg; copper 2,000 mg/kg; ferro 12,500 g; manganese 17,500 g; zinc 12,500 g; iodine 300 mg; selenium 50 mg.

2) Butylhydroxytoluene.

**Table 2:** Nutritional requirement of Japanese quail during the laying phase according to Silva et al [17]

Item	
Metabolizable energy (kcal/kg)	2,850
Crude protein (%)	20.0
Digestible lysine (%)	1.05
Digestible methionine+cystine (%)	0.72
Digestible threonine (%)	0.73
Digestible tryptophan (%)	0.2
Digestible valine (%)	0.94
Calcium (%)	2.95
Available phosphorus (%)	0.30
Sodium (%)	0.14

**Table 3:** Dilution levels and metabolizable energy used in the Japanese quail during the laying phase assay

Item	Levels of metabolizable energy (kcal/kg)								

	1,500	1,890	2,060	2,230	2,400	2,730	2,960	3,070	3,250
High	-	22.29	32.00	41.72	51.43	70.29	83.43	89.71	100
Low	100	77.71	68.00	58.28	48.57	29.71	16.57	10.29	-
Total	100	100	100	100	100	100	100	100	100

**Table 4:** Means (±standard error) of the responses and mortality of Japanese quails during the laying phase when subjected to different dietary concentrations of metabolizable energy

Levels kcal/kg		FI (g/bird·d)	EP (%/bird/d)	EW (g)	EO (g/d)	FCR (g/g)	BW (g)	ΔBW (g/bird·d gain)	MOR

Calculated	Analyzed								
1,500	1,609	25.7±2.2	2.3±1.3	10.8±0.8	0.2±0.1	98.1±38.6	53±6	−0.57±0.15	8
1,890	1,740	23.9±1.4	10.1±1.4	10.3±0.6	1.0±0.2	23.2±3.5	155±8	−0.35±0.08	1
2,060	1,895	23.5±1.3	19.1±3.9	10.8±0.4	2.1±0.4	12.0±2.8	166±5	−0.25±0.04	4
2,230	2,260	24.4±0.7	29.4±12.6	10.2±0.4	3.0±1.5	8.7±2.4	169±6	−0.16±0.05	1
2,400	2,394	24.2±0.5	50.0±16.2	10.8±0.4	5.4±1.6	4.5±1.0	175±13	−0.15±0.07	2
2,730	2,643	23.8±1.9	69.4±6.3	11.2±0.2	7.8±0.8	3.2±0.2	180±14	−0.04±0.06	1
2,960	2,892	23.5±0.7	73.9±2.2	11.2±0.3	8.2±0.4	2.9 ±0.1	181±9	0.01±0.07	1
3,070	3,045	23.4±0.7	75.1±6.2	11.3±0.3	8.5±0.8	2.8±0.2	190±8	0.08±0.02	0
3,250	3,058	23.3±0.6	77.8±3.3	11.7±0.6	9.1±0.3	2.6±0.1	194±12	0.17±0.02	0
Average		24.0	45.2	10.9	5.0	17.6	163	−0.14	2
SEM		0.103	4.176	0.066	0.483	4.251	-	0.031	-
p-value^1)^		0.026	0.001	0.001	0.001	0.001	-	0.001	-

FI, feed intake; EP, egg production; EW, egg weight; EO, egg output; FCR, feed conversion ratio by egg output; BW, body weight; ΔBW, body weight gain; MOR, mortality, per unit; SEM, standard error of the mean.

1) Probability F analysis of variance.

**Table 5:** Parameters fitted for the responses of Japanese quails during the laying phase when subjected to different concentrations of metabolizable energy in the diet

Parameters	FI (g/bird·d)	EP (%/bird/d)	EW	EO (g/d)	FCR (g/g)	ΔBW (g/bird·d gain)
ψ, Asymptote, Y axis	23.4±0.43	74.0±2.09	10.4±0.14	8.4±0.34	3.1±0.49	0.007±0.0007
α, Slope	−0.9±0.53	58.2±4.40	1.9 ±0.21	5.9± 0.41	−25.2±5.23	−0.317±0.075
τ, Break point, X axis	3,040±72	2,820±7	1,802±180	2,960± 8	2,610±87	2,892±282
β, Slope	-	-	0.78±0.12	-	-	-
p-value^1)^	0.050	0.050	0.050	0.050	0.050	0.050
R^2^	51.85	90.59	30.21	86.49	89.86	25.32

1) Probability F regression.

FI, feed intake; EP, egg production; EW, egg weight; EO, egg output; FCR, feed conversion ratio by egg output; BW: body weight; ΔBW, body weight gain.

**Table 6:** Average (±standard error) of morphological and histological parameters of the liver and oviduct of laying quails subjected to different levels of energy

Levels (kcal/kg)		BW (g)	LW (g)	LL	RLW (g)	LLW (g)	OW (g)	OL (cm)	NMC	NIC

Calculated	Analyzed									
1,500	1,609	145±19	2.4±0.1	3.7±0.1	1.2±0.2	0.7±0.1	0.5±0.2	6.4±4.4	11.0±0.1	18.0±0.1
1,890	1,740	161±80	5.0±0.9	4.9±0.7	3.0±0.5	1.9±0.5	5.5±1.3	24.6±7.7	14.2±1.2	13.6±1.8
2,060	1,895	164±15	4.7±1.2	4.1±0.4	2.8±0.5	1.8±0.5	5.3±0.8	29.5±2.6	14.8±2.2	15.6±0.8
2,230	2,260	169±17	5.2±1.3	4.1±0.3	3.0±0.8	1.9±0.3	5.6±1.1	28.7±2.4	13.0±1.1	13.8±1.2
2,400	2,394	170±16	5.3±1.0	4.6±0.4	2.8±1.0	2.4±0.4	6.4±0.7	33.0±2.7	14.2±1.5	14.7±1.5
2,730	2,643	174±10	4.2±1.4	3.5±0.7	2.5±0.9	1.8±0.8	5.2±1.6	28.9±3.3	13.2±1.1	14.0±1.4
2,960	2,892	178±80	4.8±0.7	4.3±0.2	2.8±0.4	1.9±0.3	6.3±0.9	34.0±2.8	14.8±0.7	13.4±1.5
3,070	3,045	180±21	5.6±0.5	4.1±0.2	3.4±0.4	2.2±0.4	7.1±1.2	29.2±5.6	12.8±2.7	12.6±1.8
3,250	3,058	182±13	6.6±0.4	4.3±0.3	3.8±0.3	2.6±0.4	7.5±1.3	31.5±4.0	15.0±0.6	14.4±1.8
Average		169	4.9	4.2	2.8	1.9	5.5	27.3	13.7	14.5
SEM		1.57	0.16	0.06	0.10	0.07	0.28	1.13	0.18	0.21
p-value		NS^1)^	NS	NS	NS	NS	NS	NS	NS	NS

BW, body weight; LW, liver weight; LL, liver length; RLW, right lobe weight; LLW, left lobe weight; OW, oviduct weight; OL, oviduct length; NMC, number of magno crimps; NIC, number of isthmus crimps; SEM, standard mean error.

1) NS, non-significant, p>0.05.

**Table 7:** Means (±standard errors) for number of steatosis cells, number of Kupffer cells, steatosis area, and Kupffer cell area of Japanese quail during the laying phase

Levels (kcal/kg)		Steatosis number	Kupffer’s cells number	Steatosis area (μm^2^)	Kupffer’s cells area (μm^2^)

Calculated	Analyzed				
1,500	1,609	36.16±30.67	4.83±1.00	4.54±1.36	6.15±0.11
1,890	1,740	31.86±29.26	6.94±1.79	5.70±1.38	6.81±0.20
2,060	1,895	22.63±15.90	5.80±2.19	5.06±1.37	6.83±0.25
2,230	2,260	41.33±31.50	5.40±3.19	4.74±1.01	6.43±0.24
2,400	2,394	35.79±28.70	6.93±2.71	5.48±1.31	6.57±0.25
2,730	2,643	19.83±12.67	6.63±3.59	5.27±1.94	6.46±0.32
2,960	2,892	15.12±17.23	6.60±0.79	4.79±0.88	6.59±0.25
3,070	3,045	16.87±7.88	6.03±1.02	4.80±0.54	6.49±0.20
3,250	3,058	77.80±4.82	6.80±3.01	6.95±2.15	6.68±0.65
Average		33.04	6.22	5.26	6.56
SEM		2.62	0.10	0.10	0.03
p-value		NS	NS	NS	NS

SEM, standard mean error.

NS, non-significant, p>0.05.

**Table 8:** Analysis of morphological and histological parameters of the liver and oviduct of laying quails subjected to different levels of energy

Variables	F1	F2	F3	F4
Liver weight	**0.961** ^*^	0.089	0.050	0.013
Liver length	0.062	−0.118	**0.732** ^*^	−0.135
Right lobe weight	**0.765** ^*^	−0.137	0.006	−0.002
Left lobe weight	**0.754** ^*^	0.286	0.009	0.046
Oviduct weight	0.130	**0.756** ^*^	0.320	0.002
Oviduct length	−0.006	**0.830** ^*^	−0.163	−0.017
Number of magno’s crimp	0.049	0.143	**0.795** ^*^	0.163
Esteatosis number	**0.697** ^*^	−0.237	0.189	−0.280
Esteatosis area	0.344	−0.269	0.274	**−0.761** ^*^
Kupffer cells area	0.206	−0.234	0.281	**0.816** ^*^
Explained variance	2.751	1.589	1.492	1.372
p-value^1)^	0.148	0.500	0.148	0.937

F1–4, factor.

* Significance variables within each factor.

1) The factor coefficients in bold were used for interpretation.

Probability F multivariate analysis of variance for nitrogen-corrected apparent metabolizable energy (MEn) levels.
